# *Anopheles stephensi*: a guest to watch in urban Africa

**DOI:** 10.1186/s40794-022-00165-7

**Published:** 2022-04-01

**Authors:** Eliningaya J. Kweka

**Affiliations:** 1 Department of Medical Parasitology and Entomology, Catholic University of Health and Allied Sciences, Mwanza, Tanzania; 2 Division of Livestock and Human Diseases Vector Control, P.o. Box 3024, Arusha, Tanzania; 3 Current: Tanzania Plant Health and Pesticides Authority, P.O. Box 3024, Arusha, Tanzania

**Keywords:** Malaria, Insecticide, Resistance, Habitat types, Breeding sites

## Abstract

Malaria vector control programs in Sub-Saharan Africa have invested many efforts and resources in the control of eight-sibling species of *Anopheles gambiae* complex and *An. funestus* group. The behaviour of sibling species of these vectors is well known and used for implementing the current intervention tools. The reports of *An. stephensi* in urban Africa with different habitats breeding behaviour is an alert on the success of malaria vector control efforts achieved so far. This communication intends to give an insight on what should be considered as a challenge for the management of *An. stephensi* in urban Africa to retain the achievement attained in malaria control.

## Background

Malaria vectors have been managed well for the past two decades with significant progress in preventing malaria and related adverse outcomes [1]. From 2018 to 2019 the malaria mortalities have been stalled with an increase in 2020, the efforts done so far through the distribution of long-lasting insecticidal nets (LLINs), indoor residual spray (IRS) and urban larval source management have increased the coverage [1, 2]. The gradual changes in land use, interventions and climate changes have led to species shift and re-distribution [3–6].

For a decade now in different countries of Africa there are reports of *An. stephensi* invasion [7–9]. This vector has been for long a malaria vector in south-eastern Asia [10]. The countries reported having *An. stephensi* are Djibouti, Ethiopia, Sudan and Somalia [9]. These reports have been confirmed after the DNA molecular analysis [11]. *Anopheles stephensi* is quite different from *An. gambiae* s.l. (Table 1). This species invasion has prompted the author to make a commentary on *An. stephensi* in urban Africa and its control challenges.

**Table 1:** Differences between *An. gambiae* s.l. and *An. stephensi*

Factor	Differences	Comment
Oocyte prevalence	If both feed in the same infected blood meal source *An. stephensi* have higher oocyst development rate than *An. gambiae* s.s [12].	This means *An. stephensi* are more susceptible to parasites than An. gambiae s.l.
Breeding sites	*An. stephensi* breeds in containers and water cans indoors and outdoors while *An. gambiae* s.l. breeds in the natural habitats away from human dwellings	*An. stephensi* has advantage of transmissions of malaria based on breeding sites and man access point.
Feeding and Resting preferences	*An. stephensi* higher densities are found cattle sheds than human dwellings while for *An. gambiae* s.l. feeding and resting depend on species. *An. stephensi* rests both indoor and outdoor while An. gambiae s.l. depend on the species. Most of *An. stephensi* feed on cattle while *An. gambiae* s.l. depends on species	The feeding and resting behaviour of *An. stephensi* suggests having contribution to malaria transmission for been in contact with man either indoor or outdoor

## Main text

The introduction of *Anopheles stephensi* in African countries from Asia has alerted the national malaria control programmes in re-designing vector control strategies. The author indicates the main factors which are expected to be challenges in the efforts to control the species. These challenges are;

(i) *An. stephensi* is different from the current malaria vectors available in Africa with its breeding habitats mostly utilizing containers, holes in trees, water storage tanks and roof gutters used by *Aedes aegypti* species [13] (Table 1). Also, they were found to co-habit with culicine species in polluted habitats [13]. In Sri Lanka the *An. stephensi* has been found colonizing large water bodies breeding sites [14] which for larviciding are difficult to attend effectively. This vector possess a risk of occurrence in more countries Africa as a first case was reported in Djibouti in 2012 [15], Ethiopia in 2016 [16] and in Sudan 2019 [17]. The distribution rate of *An. stephensi* is very high covering a long distance Djibouti to Sudan in 6 years.
(ii) nsecticide resistance has been reported as the main challenge for insectides used in IRS and in LLINs for other documented existing vector species [18]. In *An. stephensi,* the insecticides resistance has been reported in Sudan and Ethiopia [8, 19, 20]. Insecticides resistance confirmation is important for the vector control insectides based tools selection.
(iii) The *An. stephensi* in Asia do feeding on human and bovines, resting indoors and outdoors [12]. Due to variations on host availability in Africa it’s not well known in which host apart from humans shall feed on. The *An. stephensi* resting and feeding behaviour in all reported areas has not been yet established in African countries.
(iv) Monitoring of anthropogenic factors. Due to high rural-urban migration areas in sub–Saharan Africa, the emerging of urban agriculture, unplanned settlements, and poorly organized drainage systems effective habitats have been created [21–24]. The new species of *An. stephensi* is well known to be urban and peri urban malaria vector.

## The way forward

(i) To strengthen the entomological surveillance system with the ability to capture the presence of this invasive *An. stephensi* mosquitoes.
(ii) To coordinate capacity building for laboratory and field entomologists in identification of *An. stephensi*. This is of priority to ensure sustainacy of achieved malaria vector species control and cases in two decades, 2000 to 2020.
(iii) To establish the continuous monitoring of insecticide resistance profile of *An. stephensi* where the species will be reported to avoid impairing the existing tool efficacy.
(iv) To identify the potential breeding habitats for *An. stephensi* in urban and peri urban for appropriate control design.
(v) To establish the sentinel sites for continues data collection in all zones. These sentinels’ sites should operate on proposed standard operating procedures for species sampling, identification and insecticides resistance status.
  v) To emphases on the use of personal protection tools such as repellents for protection outdoors.

## Conclusion

The NMCPs of sub-Saharan Africa have been awaken on insuring that, the attained malaria control efforts are not compromised by the new invasive species. The way forward plans should be considered for proper management and control of this new species vector.

## Data Availability

Not applicable.

## Abbreviations

IRS: Indoor residual spray
LLINs: Long lasting insecticidal nets
NMCP: National malaria control program
WHO: World health Organisation
